# Validated tools used to assess musculoskeletal injuries in competitive rhythmic gymnasts: A systematic review

**DOI:** 10.1097/MD.0000000000043672

**Published:** 2025-08-01

**Authors:** Fei Tan, Bingquan Luo, Yang Gao, Yi Shi, Luyao Ma

**Affiliations:** a School of Physical Education and Sport Science, Fujian Normal University, Fuzhou, China; b School of Sport Management and Communication, Capital University of Physical Education and Sports, Beijing, China.

**Keywords:** assessment tool, injury surveillance, review, rhythmic gymnasts

## Abstract

**Background:**

Rhythmic gymnasts need years of hard work. However, despite their endeavor, they often face a high risk of injury due of the demands of strength, endurance, and apparatus exercises. Standardized and validated assessment tools are essential for the effective detection and treatment of these injuries. This study aimed to identify studies that utilizes validated tools to assess musculoskeletal injuries in rhythmic gymnasts, focusing on describing the content and measurement quality of the tools used.

**Methods:**

This systematic review is registered at Inplasy (no. 202530028)WOS, PubMed, Cochrane Library, China Wan Fang Data Knowledge Service Platform, and SPORTDiscus were searched to find the literature by 2 reviewers.

**Results:**

From the 174 studies screened, 21 studies included. Eleven were self-reported injury questionnaires, 3 were imaging exams, and 7 were medical records. Thirteen injury assessment tools were validated for rhythmic gymnastics.

**Conclusion:**

Valid, reliable, and specific tools to assess rhythmic gymnastics injuries are generally lacking. To increase the rigor of future injury assessment in rhythmic gymnastics, it is recommended that future studies incorporate validated sports injury systems and tools that have already been evaluated.

## 1. Background

Rhythmic gymnastics is recognized as “ballet on the carpet.” Athletes need a lot of practice and repetition, years of hard training to enhance their competitive level. In rhythmic gymnastics, girls fight for perfection. Musculoskeletal pain or discomfort is a joint clinical presentation in rhythmic gymnasts. The gymnasts perform a lot of elements in an exercise that demand the backbone exaggeratedly at the lumbar spine (dynamic elements with and without rotation, pirouettes, balance elements), elements that require the knee joint (during pirouettes), and the shoulder joint (difficult grips outside the visual range). In the face of fierce global competition, difficulty becomes the key to obtaining excellent results. In a study of 77 Chinese rhythmic gymnasts girls, 63 of them showed 179 cases of injuries in different body parts such as waist, foot, hip, shoulder, and neck.^[1]^ Whereas the injured tissues are prone to scarring, the limited ability of tissue self-repair can lead to repetitive injuries, and the problem of treatment and rehabilitation of long-term sports injuries will not only shorten the athletes’ athletic careers, but will also have a long-term impact on the health and psychology of the athletes after they retire from competition.^[2–6]^

Aim of this study is to focus on describing the content of the tools used to help rhythmic gymnastic athletes effectively identify musculoskeletal injuries. Pain management requires accurate and thorough pain assessment. The most important validity of pain assessment is its ability to track athletes’ overuse injuries. With a view to informing the development and rationalization of the selection of assessment tools, the review discuss the current status of their application and the limitations of their measurements. The transition from long practice to great athlete is hard, especially in the rhythmic gymnastics area. Athletes always have to face challenges and uncertainty, the use of standardized methods to assess injuries is an essential starting point.

## 2. Methods

This systematic review was conducted in accordance with the PRISMA checklist version 202012 and has been registered in Inplasy (registration number: 202530028). All analyses were based on previous published studies, thus no ethical approval and athlete consent are required.

### 2.1. Search strategy

A literature search for studies was conducted in PubMed, Web of science, Cochrane Library, China Wan Fang Data Knowledge Service Platform, and SPORTDiscus. The search covered articles published from inception until March 1, 2025 and was screened by 2 independent reviewers. The search terms used are detailed in Table 1 and were combined with the Boolean operator AND.

**Table 1:** Terms used for search strategy.

Theme	Search terms
Injury	“Rhythmic gymnastics” + “injury” or “rhythmic gymnastics” + “arms injure” or “rhythmic gymnastics” + “knee injure” or “rhythmic gymnastics” + “ankle injure” or “rhythmic gymnastics” + “foot injure” or “rhythmic gymnastics” + “low back pain” or “rhythmic gymnastics” + “back injury” or “rhythmic gymnastics” + “shoulder injuries” or “rhythmic gymnastics” + “softtissue injury” or “rhythmic gymnastics” + “tendon injury” or “rhythmic gymnastics” + “musculoskeletal injuries”

### 2.2. Study selection

Articles were included if they: (a) Assessed musculoskeletal injuries in rhythmic gymnastics using an assessment tool. (b) All participants were artistic gymnasts, including elite, subelite, and club levels. (c) Articles were written in English or Chinese without restriction on publication year. Articles were excluded if they were: (a) Books, conferences, abstracts, review articles, and case study designs. (b) Literature is descriptive such as reviews. If complete articles were not available, the corresponding authors were contacted. The selection of studies was performed by 2 independent reviewers. After excluding duplicates, the reviewers screened titles and abstracts according to the specified inclusion and exclusion criteria. All discrepancies were resolved by a third reviewer (Doctor of Athletic Training in Rhythmic Gymnastics).

### 2.3. Data extraction

Two independent reviewers performed standardized data extraction for all included studies, including the following: study design, sample characteristics (sample size, age, and training level), injury definition, injury assessment tools, and by whom the diagnosis was made. After identifying the tools used in the included studies, a new data extraction table was created describing the content of these tools (Table 1). The extracts were as follows: tool name and abbreviation, brief description, content framework, tool type, target population, and measurement characteristics (content validity, criterion validity, reliability, and consistency).

### 2.4. Psychometric properties definition

Reliability refers to the consistency of the results of repeated tests, and the reliability of an instrument is evaluated using common methods of estimating reliability coefficients from classical measurement theory, such as Cronbach alpha coefficient for internal consistency. The internal consistency test is used to measure the correlation of the items in the questionnaire. Cronbach alpha is between 0.70 and 0.75, which indicates a strong consistency between the items. Validity refers to the extent to which the results of an assessment are explained by empirical and theoretical support. It includes 3 aspects: content validity, structural validity, and statistical validity. Content validity ensures that the questionnaire instrument accurately captures the expectations of the target. Structural validity usually verifies whether the measurement instrument truly reflects the intrinsic structure of the model, and calibration correlation validity is usually related to the evidence of the relationship of other variables.

### 2.5. Methodological quality assessment

Methodological quality was assessed using the adapted checklist proposed by Bates and Alexander with a maximum score of 18.^[7,8]^ Studies with scores >67% (12 or more points) were categorized as high-quality studies, 33% to 67% (7–11 points) were categorized as moderate quality studies, and <33% (6 or fewer points) were categorized as low quality studies. Methodological quality was assessed by 2 independent reviewers and all discrepancies were resolved by a third reviewer.

## 3. Results

**Figure 1.**
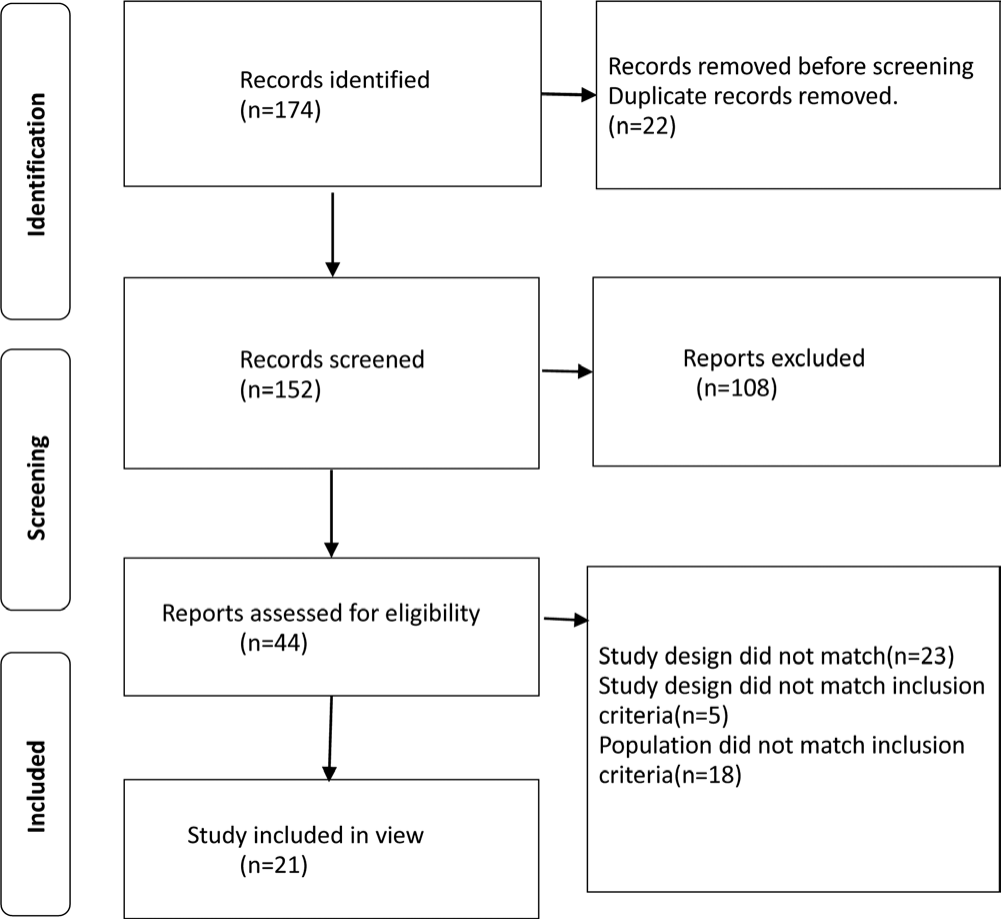
PRISMA flow diagram for the studies identification and screening process.

From the 174 studies found in the databases, 44 were screened for inclusion criteria, resulting in 21 studies included in the review that have been published in the Sport science literature using tools for musculoskeletal injury assessment (Fig. 1).

### 3.1. Sample characteristics

The 21 included studies comprised a sample of 1422 athletes, with 15 studies (71%) evaluating national level/elite athletes and 7 studies (33%) analyzing club level athletes. The results of the studies are summarized in Table 2.

**Table 2:** Characteristics of included studies.

No.	Study	Design	Sample	Injury assessment tool	Reported by	Injury definition	D&B
1	Marte Charlotte Dobbertin Gram^[9]^	Prospective study	107F, national level, age: 14.5 ± 1.6 yr, height: 162.6 ± 7.0, weight: 50.1 ± 8.7, years of training: 7.5 ± 1.9	OSTRC-H2, Beighton score, TSRQ	Self-reported	Injury location and mechanism, illness symptoms, date and time-loss and medical attention	14 (74%) high
2	Cupisti, A^[10]^	Prospective controlled study	70F, subelite level, age: 14.7 ± 2 yr, height: 1.6 ± 0.07; weight: 47.6 ± 7.4	Injury record booklet	Self-reported	Anatomical site,type and circumstance of injury medical intervention,number of full training	10 (53%) medium
3	Manuel Sabeti^[11]^	Prospective study	144F, elite level, age: 15.24 ± 2.39 years, height: 162.84 ± 7.59, weight: 44.67 ± 6.57, month of pain: 2.29 ± 3.01	A questionnaire designed by the authors, visual analogue scale	Self-reported	Painful body region, the number of weeks or months of pain, the kind of treatment, and absence from training or competition	11 (58%) medium
4	Marte Charlotte Dobbertin Gram^[12]^	Randomized controlled trial	205F, national and international level, age: 13.8 ± 2.0, height: 161.3 ± 7.6, weight: 50.0 ± 9.2, h/wk training load: 15.2 ± 6.2	OSTRC-H2	Self-reported	Monthly prevalence of substantial overuse injuries in the knees,lower back,hip/groin and calf/ankle/foot	14 (74%) high
5	Merrilee N Zetaruk^[13]^	Retrospective study	20F, national level, age: 14.8–18.8 yr, year of training: 6.5 ± 2.8, h/wk training load: 26.2 ± 7.5	Reported morbidity survey	Self-reported	All complaints injury (minor,moderate or major), and time-loss	12 (63%) medium
6	Odysseas Paxinos^[14]^	Retrospective study	73F, elite level, Olympic, world Game and European Championship medalists, age: 20.5 ± 3.42, h/d training: 3, during preparation game 8–12 h/d	Medical records	By the team physician or physiotherapists	Symptoms,anatomic area affected, injury mechanism and treatment	8 (42%) medium
7	Reeti Gulati^[15]^	Retrospective study	79F, highest competitive level (9, 10 or elite/national level), age: 14.61 ± 2.61, years of training: 9.8 ± 2.7	Electronic medical record	By the team physician	Diagnosis, mechanism of injury (acute vs overuse), injury type (e.g., joint sprain, muscle strain, stress fracture), body part injured, and injury setting (practice vs competition)	8 (42%) medium
8	Miletic A^[16]^	Cross-sectional study	28F, national level, age: 11.85 ± 2.58; height: 149.53 ± 12.86, weight: 37.89 ± 10.99, BMI: 16.54 ± 2.12, h/wk training load: 12	SEFIP questionnaire	Self-reported	Any pain, injury body regions, intensity of pain	9 (47%) medium
9	Renan Codonhato^[17]^	Quantitative and qualitative study	8F, national level, age: 20.4 ± 2.5	Physical therapy records	By the team physiotherapist	Injury severity (minor, mild and severe) and injury duration, time-loss	10 (53%) medium
10	Dartagnan Guedes^[18]^	Retrospective study	236F, young gymnasts practice for at least 2 yr, age: 13.4 ± 1.9, height: 155.7 ± 7.8, weight: 45.8 ± 7.6, years of training: 5.6 ± 2.7, h/wk training load: 18.4 ± 7.5	A questionnaire designed by the authors	Self-reported	Any pain or discomfort include type (muscle/tendon, joint/ligament and bone), location (spine, upper and lower extremities), occurrence event (training and competition) and posttreatment return conditions (symptomatic and asymp tomatic)	12 (63%) medium
11	Cupisti A^[19]^	Cross-sectional study	67F, club level, age: 14.7 ± 2.0, height: 1.60 ± 0.07, weight: 47.6 ± 7.4, h/wk training load: 14.1 ± 4.1	A standardized physical examination protocol designed by Finnish Institute of Occupational Health	Self-reported	Low back pain symptom, pain location,Intensity of pain	9 (47%) medium
12	Oltean Antoanela^[20]^	Cross-sectional study	30F, age: 11–13, junior to third category, years of training: 6.5 ± 2.7, h/wk training load: 3	A questionnaire designed by the authors	Self-reported	Injure area, moment of injury, movement caused the injury, diagnosis, treatment	10 (53%) medium
13	Kim Chanwoo^[21]^	Cross-sectional study	25F, college students, national level age: 21.09 ± 1.14, height: 163.95 ± 4.06, weight: 49.21 ± 4.95, years of training:11.36 ± 1.39	Daily injury reports form of International Olympic committee	Medical team	Any pain, injury region acute or chronic injuries	12 (63%) medium
14	Hobson A^[22]^	Prospective study	10F, club level, age: 15.80 ± 2.04, years of training: 5.60 ± 0.84, h/wk training load: 8.70 ± 4.66	A questionnaires designed by reviewing the Ontario Gymnastics Injury Study of 1988,and the university of Saskatchewan Gymnastics study of 1991	Self-reported	Injury body part,type of injury, timing of injury, cause of injury, missed training, current health status	13 (68%) high
15	Paula Barreiros Debien^[23]^	Case study	6F, elite level, age: 21.0 ± 7.1, height: 1.64 ± 0.09, weight: 53.0 ± 8.0, years of training: 14.8 ± 5.8	A individual report	By the medical staff	Injury body region, injury type, time-off	8 (42%) medium
16	Kristina Hassmannová^[24]^	Cross-sectional study	22F, elite level, age: 13.52 ± 1.24	A questionnaire designed by the authors	Self-reported	Any pain, injury region, professional treatment	11 (58%) medium
17	Boštjan Jakše^[25]^	Cross-sectional study	17F.national level, age: 17.4 ± 4.1, height: 159.8 ± 6.2, weight: 54.8 ± 5.3, h/wk training load: 23.5 ± 1.4, years of training: 10.5 ± 3.4	OSTRC	Self-reported	Any pain, injury location, injury status	10 (53%) medium
18	Subash C Jha^[26]^	Cross-sectional study	1F, national level, age: 17	MRI	Musculoskeletal radiologists	Lesions visible on MRI	6 (33%) low
19	Tanchev, Panayot I. MD^[27]^	Cross-sectional study	100F, club level, age: 12.44 ± 1.65, years of training: >5	Radiograph	Musculoskeletal radiologists	Lesions visible on MRI	10 (53%) medium
20	Zhang Jing^[28]^	Cross-sectional study	114F, club and national level, age: 18.30 ± 2.49, height: 162.50 ± 3.72, weight: 39.70 ± 1.49, years of training: 9.70 ± 1.64	Radiograph	Musculoskeletal radiologists	Lesions visible on MRI	9 (47%) medium
21	Hu Xiao-Fang^[29]^	Cross-sectional study	60F, national level, age: 9–14, years of training: 4–8	A questionnaire designed by the authors	Self-reported	Any pain, injury region	9 (47%) medium

### 3.2. Description of the identified tools

Among the included 21 studies, 19 instruments were identified to assess musculoskeletal impairments (Table 2). Two were imaging exams, 10 were self-reported injury questionnaires, 7 were medical records or incidence reports, and 6 studies did not report the measurement characteristics of the instruments. Table 3 lists a summary of information retrieved from the original validation studies.

**Table 3:** Full tiles of injury assessment tools.

Acronym	Meaning
OSTRC-H2	Oslo Sports Trauma Research Centre Questionnaire on Health Problems
TSRQ	Triad-Specific Self-Report Questionnaire
VAS	Visual Analog Scale
SEFIP	Self-Estimated Functional Inability because of Pain questionnaire
BS	The Beighton score

### 3.3. Methodological quality assessment

Following the adapted Downs and Black’s checklist, 3 studies (14%) achieved high-quality score,^[9,12,22]^ and 17 studies (80%) reported a medium quality.^[10,11,13–21,23–25,27–29]^ One of the included studies^[26]^ showed scores lower than 33%.

### 3.4. Imaging exam

Imaging exam are commonly used in assess injuries, it usually includes X-ray imaging, MRI, and ultrasound imaging. Imaging exam were used by 3 studies in this review.^[26–28]^ One of which was a CT imaging study and 2 of which were X-ray imaging studies. Because a large number of skills in rhythmic gymnastics rely on over-amplitude flexion, extension, or torsion of the spine, athletes often suffer from spinal hypermobility in sports, and all 3 studies focused on spinal injuries. One study examined stress fractures of the thoracic spine in elite artistic gymnasts, one examined scoliosis in athletes, and one focused on injury characteristics of the lumbar segment of the spine. All 3 studies were conducted by medical professionals, 2 used standardized physical examination protocols, and one performed height and weight measurements. CT imaging and X-ray imaging have shown good reliability in the assessment of injury in rhythmic gymnasts. But no studies have analyzed the intra-rater reliability of 2 physicians, and unfortunately, once the athlete has been imaged radiologically, it is clear that the athlete has suffered a serious injury which will Impact on their athletic careers.

### 3.5. Medical records

Medical records were used by 5 studies in this review.^[14,15,17,21,23]^ Evaluation by physicians or medical team can directly support injury care for the athletes. These are evaluated through a case-by-case injury assessment, where the clinician gives a specialized evaluation and sports medicine diagnosis, and is able to provide systematic medical action for the athlete.

### 3.6. Self-report on injury prevention behavior

The injured self-report was includes questionnaires and injury records. Questionnaires were used by 11 out of the 21 studies included in this review,^[9,11,12,16,18–20,22,24,25,29]^ three of them used the Oslo Sport Trauma Research Center Overuse Injury Questionnaire (OSTRC),^[9,12,25]^ and one of which incorporated the Beighton score and Triad-Specific Self-Report Questionnaire, which measure the athlete’s validity of systemic overactivity and autonomic conditioning. One of the study used the Self-Estimated Functional Inability because of Pain (SEFIP),^[16]^ one used a self-administered questionnaire and a visual analog scale for the assessment of pain perception,^[11]^ and one used a standardized physical examination protocol designed by the Finnish Institute of Occupational Health to collect information on general impairments.^[19]^ The remaining 5 questionnaires were individually developed questionnaires, with the exception on of one which questionnaire design using a review of the 1988 Ontario Gymnastics Injury Study and the 1991 University of Saskatchewan Gymnastics Study, the other questionnaires lacked specific validation studies and designation names, although they were designed by researchers with relevant backgrounds. Two studies used the injury record/morbidity survey.^[10,13]^

#### 3.6.1. Type of tool

Martin et al^[30]^ categorized the tools into 3 types, including generic measurement tools, region-specific measurement tools, and disease-specific measurement tools. From the 9 self-reported questionnaires, 8 (88%) were generalized measurement tools, includes OSTRC, SEFIP and self-developed questionnaires,^[9,11,12,16,18,20,22,24,25,29]^ which measured systemic pain in low back, ankles, knees, shoulders, hips, necks, and so on. Two studies that used the injury record/morbidity survey also addressed the whole-body range of athletes’ records^[10,13]^ and one (12%) of the questionnaires measuring pain in low back designed by the Finnish Institute of Occupational Health, there is no questionnaire design to measure disease-specific disorders in rhythmic gymnasts.

#### 3.6.2. Target population

Nine of the 11 questionnaires were originally validated for athletes.^[9,11,12,18,20,22,24,25,29]^ One was designed to assess the injury of dancers (SEFIP).^[16]^ One designed to be administered to the general population.^[19]^ The injury record/morbidity surveys were all sports medicine oriented for professional athletes.

#### 3.6.3. Length

The questionnaires ranged from 4 to 40 items, and two of the questionnaires did not specify the number of questions to be included in the questionnaire.

#### 3.6.4. Psychometric properties

##### 3.6.4.1. Internal consistency

Four of the 11 questionnaires estimated the Cronbach alpha (SEFIP, OSTRC). One of the questionnaires (the protocol designed by Finnish Institute of Occupational Health) estimated the kappa value and ICC value. However, most self-administered questionnaires not performed the consistency test.

##### 3.6.4.2. Content validity

Three of the 11 questionnaires were designed with experts’ advice,^[19,22,29]^ OSTRC-H2 was developed based on OSTRC for over injury, SEFIP was developed based on NMQ, and the rest of the questionnaires^[11,18,20,22]^ were not subjected to content validity process.

##### 3.6.4.3. Construct validity

One of the 11 questionnaires (visual analog scale)^[11]^ specified its hypotheses in advance and demonstrated consistent results, confirming their previous hypotheses.

##### 3.6.4.4. Criterion validity

Five of the 11 questionnaires showed correlation values with other validated tools.^[9,12,16,22,25]^

## 4. Discussion

The aim of the review was to synthesize the adequacy of validated measurement tools used in musculoskeletal injury research in rhythmic gymnastics to measure injury risk in athletes, focusing on describing the content of the tools used. To our knowledge, this is the first systematic review of assessment tools used in the rhythmic gymnastics injury research literature.

### 4.1. Methodological quality assessment

The majority of studies showed moderate methodological quality, demonstrating the importance of further adoption of standardized study designs in future artistic gymnastics injury research.

### 4.2. Tools used to assess musculoskeletal injuries

It can be seen that a large percentage of studies rely on clinicians’ diagnosis. This highlights the importance of professional medical care in injury evaluation. However, it also indicates an overreliance on medical professionals for monitoring injuries in rhythmic gymnastics. Which complicates injury prevention, as medical consultations typically occur only after an injury has taken place. The use of effective simple, standardized and reliable assessment tools is rare.

This review also shows that the use of musculoskeletal injury assessment questionnaires in rhythmic gymnastics is still in the initial stage. Only a very few researchers used evaluated injury assessment tools in their studies in recent years. Most of injuries are due to over-practice, the questionnaires can be considered as efficiently tools to prevent serious injuries. However, the majority of researchers still use self-developed questionnaires, although sports injury experts were consulted of these questionnaires. Rhythmic gymnasts were not included in the questionnaire development process, it makes difficult to ensure the quality of the evaluation methodology. In order to improve the quality of the research methodology, it is recommended that future studies incorporate validated sport injury systems and tools that have already been evaluated, such as OSICS, MRI, iHOT-12, HAGOS.

### 4.3. Limitations

Despite the rigorous search strategy used, it is possible that relevant studies were missed. Another limitation arise due to the relatively small number of athletes playing rhythmic gymnastics, some of the studies had small sample sizes. Additionally, because the authors are not very proficient in Russian, almost no relevant athlete injury studies from Russia were included, which is a shortcoming. Nonetheless, the full range of injury assessment tools was included as much as possible.

### 4.4. Future research

In 2023, the IOC released a consensus statement on sports injuries. They called for consistent study designs, data collection, and terminology for detecting injuries and illnesses in golf, tennis, cycling, and para-sport. Rhythmic gymnastics should also find new ways to monitor athletes’ injuries and use standardized methods for injury and disease research. Additionally, injury surveillance needs to be better tailored for both professional and amateur athletes in rhythmic gymnastics.

## 5. Conclusion

Valid, reliable and specific tools to assess rhythmic gymnastics injuries are lacking in general. Although pain is a subjective feeling, self-report questionnaires are a recommended and valuable method for the field of rhythmic gymnastics. Researchers may consider translating the original instruments into their national language and modifying them to suit their national rhythmic gymnastics before using these scales. The use of these tools is essential to ensure accurate evaluation and improves the quality of injury detection in rhythmic gymnasts.

## Author contributions

**Data curation:** Fei Tan, Yang Gao, Yi Shi, Luyao Ma.

**Methodology:** Fei Tan.

**Writing—original draft:** Fei Tan.

**Writing—review & editing:** Fei Tan, Bingquan Luo.
